# First two unrelated cases of isolated sedoheptulokinase deficiency: A benign disorder?

**DOI:** 10.1007/s10545-014-9809-1

**Published:** 2015-02-03

**Authors:** Mirjam M. C. Wamelink, Ruben J. J. F. Ramos, Annette P. M. van den Elzen, George J. G. Ruijter, Ramon Bonte, Luisa Diogo, Paula Garcia, Nelson Neves, Benjamin Nota, Arvand Haschemi, Isabel Tavares de Almeida, Gajja S. Salomons

**Affiliations:** 1 Department of Clinical Chemistry, Neuroscience Campus Amsterdam, VU University Medical Center, De Boelelaan 1117, 1081HV Amsterdam, The Netherlands; 2 Metabolism & Genetics, iMed.UL, Fac. Pharmacy- Univ. of Lisboa, Lisboa, Portugal; 3 Department of Paediatrics, Reinier de Graaf Gasthuis Delft, Delft, The Netherlands; 4 Department of Clinical Genetics, Erasmus Medical Center, Rotterdam, The Netherlands; 5 Paediatric Hospital of Coimbra, Coimbra, Portugal; 6 Department of Laboratory Medicine, Medical University of Vienna, Vienna, Austria

## Abstract

We present the first two reported unrelated patients with an isolated sedoheptulokinase (SHPK) deficiency. The first patient presented with neonatal cholestasis, hypoglycemia, and anemia, while the second patient presented with congenital arthrogryposis multiplex, multiple contractures, and dysmorphisms. Both patients had elevated excretion of erythritol and sedoheptulose, and each had a homozygous nonsense mutation in *SHPK*. SHPK is an enzyme that phosphorylates sedoheptulose to sedoheptulose-7-phosphate, which is an important intermediate of the pentose phosphate pathway. It is questionable whether SHPK deficiency is a causal factor for the clinical phenotypes of our patients. This study illustrates the necessity of extensive functional and clinical workup for interpreting a novel variant, including nonsense variants.

**Electronic supplementary material:**

The online version of this article (doi:10.1007/s10545-014-9809-1) contains supplementary material, which is available to authorized users.

## Introduction

Sedoheptulokinase (SHPK; EC 2.7.1.14), formerly known as carbohydrate-kinase-like (CARKL), is an enzyme that phosphorylates sedoheptulose to sedoheptulose-7-phosphate (sedoheptulose-7P) (Touchman et al 2000; Wamelink et al 2008; Kardon et al 2008; Haschemi et al 2012). *SHPK* is highly expressed in liver, kidney, pancreas, and heart tissue. Sedoheptulose-7P is an important intermediate of the pentose phosphate pathway (PPP), a key pathway of carbohydrate metabolism. The PPP and glycolysis are tightly connected: The PPP is located in the cytosol and is primarily responsible for the production of cytosolic nicotinamide adenine dinucleotide phosphate, reduced (NADPH) and ribose-5P. The SHPK protein co-localizes with glucose-6P dehydrogenase in the cytoplasm (Haschemi et al 2012). SHPK seems to be an important regulatory enzyme, being responsible for feeding sedoheptulose-7P molecules to the PPP independently of glucose. Effectively, this allows the reversible function of transaldolase (TALDO) and/or transketolase when glyceraldehyde-3-phosphate, arising from the glycolytic pathway, is used by the PPP, when increased amounts of NADPH and/or ribose moieties are needed for nucleotide production (Wamelink et al 2008; Nagy and Haschemi 2013). Free sedoheptulose can be either derived dietarily from fruits and vegetables (Kardon et al 2008) or formed enzymatically by dephosphorylation from sedoheptulose-7P or possibly from other heptoses, such as mannoheptulose or 7-O-galloyl-sedoheptulose. To date, no specific sedoheptulose-7P phosphatase has been reported.

Knockdown of *SHPK* in a mouse macrophage cell line resulted in increased glyceraldehyde-3P, xylulose-5P, and ribose-5P intracellular levels. Sedoheptulose concentrations were not notably changed, while sedoheptulose-7P was significantly decreased (Haschemi et al 2012), which resulted in rerouting of glucose from aerobic to anaerobic metabolism and was accompanied by a decreased nicotinamide adenine dinucleotide (NAD)/NADH ratio. In the same cell line, the opposite was found by overexpressed *SHPK*, although sedoheptulose-7P concentrations remained normal. In a screen for new regulators of macrophage activation, overexpression of *SHPK* was found to block lipopolysaccharide (LPS)-induced tumor necrosis factor alpha (TNFα) secretion by these cells (Haschemi et al 2012). In vitro and in vivo, *SHPK* transcript is endogenously downregulated in mice and humans during LPS-induced inflammation. In vitro, knockdown of the gene by micro-RNA-adapted short hairpin RNA (shRNA-mir) results in mild activation of macrophages. This indicates that SHPK-dependent metabolic reprogramming is required for proper M1- and M2-like macrophage polarization and is a rate-limiting requirement for appropriate glucose flux during macrophage polarization (Haschemi et al 2012).

So far, SHPK deficiency has not been described in humans as an isolated defect and is only known as a combined defect, with cystinosis caused by a 57-kb deletion extending from exon 10 of *CTNS* (cystinosin, lysosomal cystine transporter), upstream through *SHPK*/*CARKL*, to intron 2 of *TRPV1* (transient receptor potential vanilloid 1) (Touchman et al 2000; Freed et al 2011). Patients with this deletion have the infantile nephropathic cystinosis type (MIM 219800), but the clinical relevance of SHPK deficiency remains unclear. In urine of these patients, high concentrations of sedoheptulose and erythritol are detected, and in bloodspots, sedoheptulose is also elevated compared with controls or patients with isolated cystinosis (Wamelink et al 2008, 2011).

We diagnosed two unrelated patients with isolated SHPK deficiency caused by different nonsense mutations in *SHPK*.

## Materials and methods

### Patients

Patient 1, a boy (currently 3 years old), was born after 35 weeks’ gestation from a healthy Caucasian mother and unknown father (suspected consanguineous relationship). The infant presented with perinatal asphyxia, birth weight of 1,750 g (P2.3–P5), craniofacial dysmorphisms (high forehead with very large fontanels, hypotelorism, and small orbits), unilateral inguinal hernia, and a sepsis-like condition with intestinal pseudoobstruction, transient hypoglycemia, cholestatic hepatitis, and transient renal failure (maximum plasma creatinine 132 μmol/L and urea 11 mmol/L at day 5 of life, which normalized on day 10). He has chronic diarrhea, with steatorrhea and severe growth retardation including short stature and macrocephaly (relative to stature). Frequent, mostly asymptomatic, short-fasting hypoglycemic episodes (with normal lactate and ketones) and postprandial hyperglycemia aggravated by the end of the first year of life persist in spite of frequent meals. Transient hypocortisolism was identified, but hypoglycemia did not respond to specific treatment. He is currently under exocrine pancreatic yeast supplementation. He has moderate intellectual disability and neurosensorial deafness. He needed several blood transfusions due to chronic microcytic hypochromic anemia, with normal ferritin, leucocytes, and platelets. Because of childhood infections (bronchiolitis and middle ear infections with febrile rhinopharyngitis), inpatient admission was needed due to difficulty in glycemic control; immune system deficiency was ruled out. His abdominal skin is strikingly thin, with upper collateral circulation and diastasis recti without liver or spleen enlargement.

**Fig. 1:**
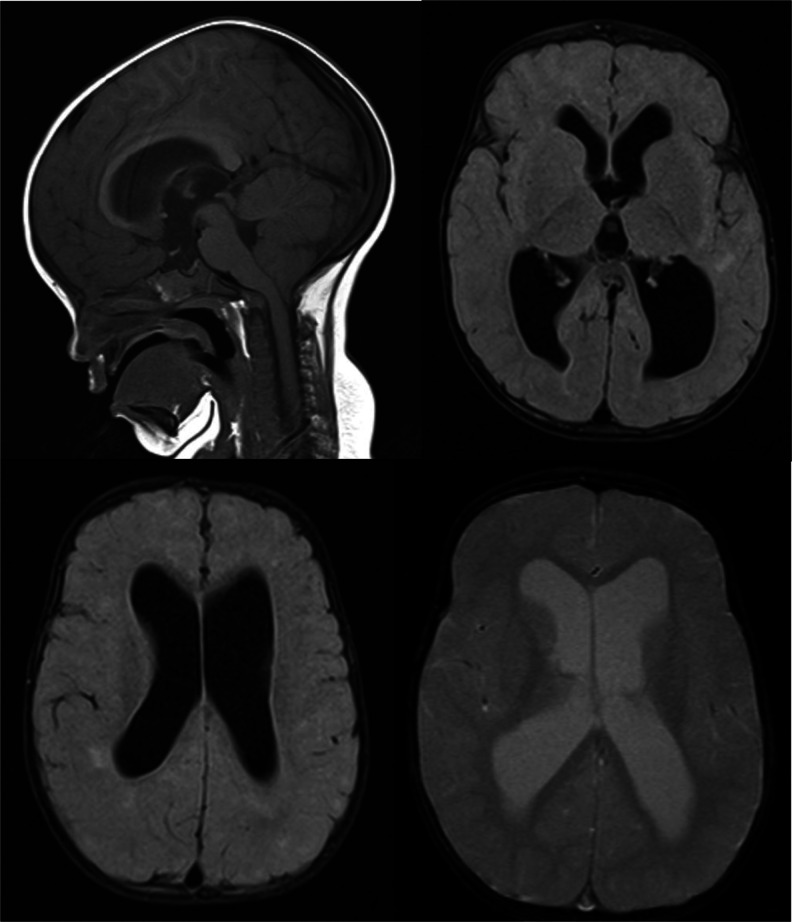
Magnetic resonance imaging (MRI) of patient 1 at the age of 3 years showing a Chiari malformation type 1, ventricular enlargement, cerebral subcortical atrophy, hyperintense periventricular lesions, and hypomyelinization

Brain magnetic resonance imaging (MRI) was normal in the neonatal period. At the age of 3 years, MRI showed ventricular enlargement, cerebral subcortical atrophy, hyperintense periventricular lesions, hypomyelinization, and a Chiari malformation type 1 (Fig. 1).

A broad etiological investigation including peroxisomal function, carbohydrate-deficient transferrin, acylcarnitine profile, urinary organic acids, microarray comparative genomic hybridization (CGH) and *UBR1*, *SBDS*, and *COX412* genes has been inconclusive. At the age of 20 months, during a metabolic workup, elevated urinary concentrations were found for erythritol and sedoheptulose with gas chromatography mass spectrometry (GC-MS). His immune system was evaluated. Immunoglobulin (Ig) G, A, M, and E levels were normal; leucocyte number and distribution and lymphocyte populations T, B, and NK were investigated; an inverted CD4/CD8 and low CD19 and CD56 were found but were not considered relevant. In the lymphoblast transformation test, there was a normal proliferative response to the mitogens phytohemagglutinin(PHA), pokeweed mitogen (PWM), and staphylococcal protein A (SPA).

Patient 2, a girl (currently 2 years old), second child of healthy consanguineous parents from Turkish heritage, was born at term. External cephalic version was successfully performed antenatally because of breech presentation. She presented at birth with perinatal asphyxia, birth weight of 2,580 g (P2.3–P5), congenital arthrogryposis multiplex, hip dysplasia, and numerous contractures. She has multiple dysmorphisms: round, asymmetrical eyes (right eye larger) with ptosis and Bells phenomenon; small mouth; high nasal bridge; dysplastic low-set ears, most prominent on the right; adducted thumbs; strikingly smooth palms; and small feet. From birth, she has had enormous feeding problems due to unsafe swallowing. Developmentally, she makes good contact but has impairments at all domains.

At the age of 7 days, her metabolic screening showed strongly elevated erythritol and sedoheptulose (GC-MS). MRI of the brain at 7 months showed no abnormalities, with symmetrical slim ventricles. Her immune system was evaluated: white blood cell differentiation, total IgA, total IgM, total IgG, and subclasses were all normal. She has not suffered from infections and is growing well. Genetic screening (array, targeted next-generation sequencing for intellectual disability) showed no abnormalities, but there is a homozygous nonsense mutation in SHPK.

### Analysis of erythritol, sedoheptulose, and sedoheptulose-7P in urine

Erythritol, sedoheptulose, and sedoheptulose-7P were quantified in urine of both patients by liquid chromatography tandem mass spectrometry (LC-MS/MS), as described (Wamelink et al 2005a, 2007).

### Molecular analysis of *SHPK* and *TALDO1*

*SHPK*, GenBank accession number NM_013276.2, is mapped to chromosome 17. The exons, including splice sites, were amplified by polymerase chain reaction (PCR). The open-reading frame (ORF) of *SHPK* and splice sites have been analyzed by DNA sequence analysis. All seven exons and adjacent splice sites of the *SHPK* gene of both patients were amplified by PCR and analyzed by direct DNA sequence analysis (for primers, see Supplementary Table 1). DNA of both patients’ mothers and one father (of patient 2) was only investigated for the amplicon containing the familial mutation. Amplicons were analyzed by capillary electrophoresis using an ABI 3130xl genetic analyzer (Applied Biosystems, Nieuwerkerk a/d lJssel, The Netherlands) and assessed using Mutation Surveyor® (Softgenetics, PA, USA). In addition to the genomic DNA (gDNA) analysis, we also investigated the *SHPK* transcript. RNA was isolated from PAXgene blood RNA tubes (QIAGEN, Benelux B.V., Venlo, The Netherlands) of patient 1 and his mother; complementary DNA (cDNA) was synthesized, and subsequently, the full-length ORF was amplified. These amplicons were directly sequenced, as described above. The reference sequence NM_013276.2 was used, and the A of the ATG translation initiation codon is nucleotide 1 following guidelines of the Human Genome Variation Society (www.hgvs.org/mutnomen).

Mutation analysis of transaldolase (*TALDO1*, GenBank accession number NM_006755.1) was performed in patient 1 by direct sequence analysis of genomic DNA (gDNA), basically as described previously (Verhoeven et al 2001).

### Analysis of SHPK activity in fibroblasts

To assay SHPK activity, fibroblast homogenates from patient 1 and simultaneously two control fibroblast homogenates were incubated with sedoheptulose and adenosine triphosphate (ATP). Incubation was performed as described earlier (Wamelink et al 2008). After incubation, sedoheptulose-7P formation was analyzed, as described (Wamelink et al 2005b) using a calibration curve of sedoheptulose-7P (Sigma-Aldrich, St Louis, MO, USA).

### SHPK Western blot analysis

The SHPK protein content of fibroblasts homogenates, untreated and treated with cyclohexamide for protein stability, from patient 1 was assessed by Western blot analysis and compared with a homogenate from control fibroblasts (untreated with cyclohexamide). Briefly, washed-cell pellets were homogenized in radioimmunoprecipitation assay (RIPA) buffer, cleared from debris by centrifugation, and 18 μg protein of each sample was resolved on a 12 % polyacrylamide gel. After blotting, SHPK protein was detected with a primary antibody against SHPK (ab69920, Abcam Inc.). Recombinant human SHPK was used to demonstrate the specificity of the SHPK antibody. Equal protein loading was indicated by beta actin (A5316, Sigma) detection.

## Results

### Analysis of erythritol, sedoheptulose, and sedoheptulose-7P in urine

Strongly elevated excretion of erythritol and sedoheptulose was detected in both patients, with low-to-normal excretion of sedoheptulose-7P, biochemically suggesting SHPK deficiency (Table 1).

**Table 1:** Metabolite concentrations in urine (mmol/mol creatinine) from the two patients with sedoheptulokinase (SHPK) deficiency compared with age-matched control ranges

Metabolite (mmol/mol creatinine)	Patient 1 (1.6 years)	Age-matched control range patient 1	Patient 2 (7 days)	Age-matched control range patient 2
Erythritol	**1,045**	76–192	**2,753**	58–162
Arabitol	**135**	52–88	**136**	27–97
Ribitol	**32**	9–24	16	7–16
Sedoheptulose	**576**	ND–9	**253**	ND–40
Sedoheptulose-7P	< Detection limit (0.02)	ND–0.1	< Detection limit (0.05)	ND–0.1

Values in bold are above the control range

*ND* not detectable

### Molecular analysis gDNA of *SHPK* and *TALDO1*

In both patients, a presumed homozygous variant was detected (patient 1: c.355C > T; p.Arg119X; patient 2: c.211G > T; p.Glu71X), both predicted to result in truncated nonfunctional proteins. The mother of patient 1 was found to be a carrier of the same mutation. DNA from the father was not available for mutational analysis. The parents of patient 2 were both carriers of the variant found in their daughter. *TALDO1* was also sequenced in patient 1 for differential diagnosis, but no pathogenic mutations were detected.

### RNA analysis *SHPK*

Reverse transcriptase polymerase chain reaction (RT-PCR) of RNA isolated from blood of the mother of patient 1 followed by sequencing of the *SHPK* transcript showed equal expression of the wild-type allele as that containing the nonsense mutation. In RNA (cDNA) of patient 1, only the *SHPK* transcript containing the nonsense mutation was detected.

### Analysis of SHPK activity in fibroblasts

In fibroblasts from patient 1, strongly reduced formation of sedoheptulose-7P was detected when compared with control fibroblasts (2.9 nmol/h/mg protein; controls 20–54), indicating SHPK deficiency.

### SHPK Western blot analysis

**Fig. 2:**
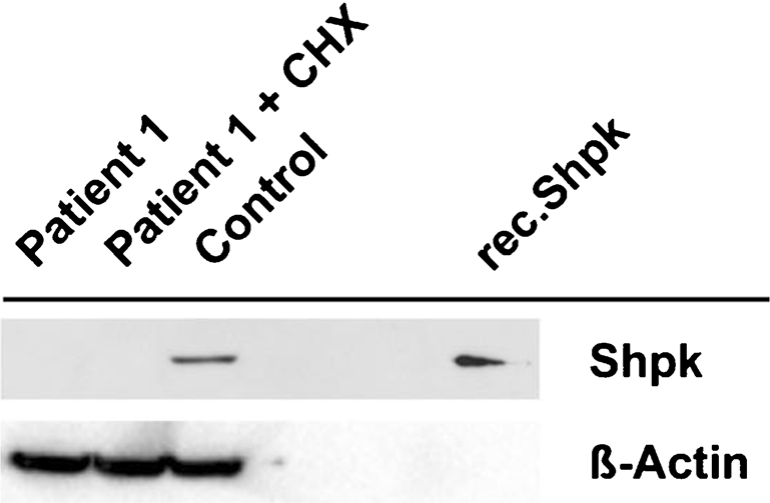
Sedoheptulokinase (SHPK) protein expression in untreated and cyclohexamide (CHX)-treated fibroblast homogenates from patient 1 and untreated control fibroblasts. Recombinant human SHPK was used to demonstrate specificity of the SHPK antibody

We detected no mature SHPK protein in untreated or cyclohexamide-treated fibroblasts isolated from patient 1. In contrast, fibroblasts derived from a control individual showed a prominent SHPK protein content (Fig. 2).

## Discussion and conclusions

We present two patients with abnormal sugar and polyol profiles (elevated excretion of sedoheptulose and erythritol), suggesting SHPK deficiency.

Clinical presentation in patient 1, with neonatal cholestasis, hypoglycemia, anemia, and dysmorphism, together with the biochemical abnormalities of elevated excretion of erythritol and sedoheptulose, raised the suspicion of TALDO deficiency (MIM 606003). However, the polyols arabitol and ribitol were only mildly elevated and sedoheptulose-7P was within the control range, while in patients with TALDO deficiency, sedoheptulose-7P in particular is highly elevated.

Patient 2 had a different clinical presentation, with congenital arthrogryposis multiplex, multiple contractures, and dysmorphisms. She has one healthy, older brother who had a normal urinary sugar and polyol profile (data not shown).

TALDO deficiency in both patients was ruled out by either biochemical or DNA studies.

Since SHPK seems to be important in macrophage polarizations, we looked for immunological dysfunction in the two patients with the isolated SHPK deficiency, and although they are still very young for a complete evaluation, they do not seem to have any obvious immune system dysfunction. SHPK seems to have a regulating function in glucose metabolism, contributing to the direct metabolic flux of cellular sedoheptulose-7P, which is a direct product and substrate of glucose metabolism. Deficiency of SHPK might therefore in part contribute to the problems in glycemic control, as seen in patient 1.

The metabolite profile in both patients was in line with our findings in cystinosis patients with the 57-kb deletion, including *SHPK* (Wamelink et al 2008). Kardon et al. indicated that the accumulation of erythritol is likely derived from sedoheptulose, which—in vitro, at least—can be converted to sedoheptulose-1-phosphate by fructokinase. The resulting sedoheptulose-1-phosphate would be converted by aldolase B to erythrose and dihydroxyacetone–phosphate, and erythrose would then finally be reduced to erythritol (Kardon et al 2008). Clinical features as described in our patients are not known in patients with infantile nephropathic cystinosis caused by the 57-kb deletion (Table 2). In general, infantile nephropathic cystinosis becomes symptomatic between the ages of 6 and 12 months, with proximal renal tubulopathy (renal Fanconi syndrome). Endocrinological, hepatic, gastrointestinal, muscular, and neurological abnormalities have also been described (Nesterova and Gahl WA. Cystinosis 2001). Mildly altered craniofacial morphology in cystinosis patients may result in swallowing difficulties and respiratory complications in the second decade (Bassim et al 2010) of life. Both of our patients presented neonatally and did not develop renal tubular Fanconi syndrome.

**Table 2:** Signs and symptoms of the two patients with sedoheptulokinase (SHPK) deficiency compared with infantile cystinosis and transaldolase (TALDO) deficiency

Symptoms	Patient 1 (3 years)	Patient 2 (1 year)	Infantile cystinosis^a^ (incl. 57 kb deletion)	TALDO deficiency
Anemia	+	−	−	+
Cardiac abnormalities	−	−	−	+
Contractures	−	+	−	−
Delayed puberty	?	?	+	+/−
Diarrhea	+	−	−	−
Facial dysmorphia	+	+	+/−	+/−
Failure to thrive	+	+	+	+/−
Feeding difficulties	−	+	+ (second decade)	−
Hypothyrodism	−	−	+	+/−
Infectious diseases	+/−	+/−	−	−
Liver disease	+ (neonatal)	−	+ (second decade)	+
Neonatal hypoglycemia	+	−	−	+/−
Intellectual disability	+	+	−	−
Retinopathy	−	−	+	−
Rickets	−	−	+	+/−
Tubulopathy	+ (renal failure)	−	+ (Fanconi syndrome)	+/−

*−* Absent, *+* present, *+/−* may occur, *?* unknown

^a^No difference in clinical phenotype between patients with other mutations causing severe infantile nephropathic type or patients with the deletion has been found (Heil et al 2001)

Isolated SHPK deficiency is confirmed by mutation analysis of the *SHPK* gene. In each of our two patients, a (presumed) homozygous nonsense variant (patient 1: c.355C > T; p.Arg119X; patient 2: c.211G > T; p.Glu71X) was detected, both predicted to result in truncated nonfunctional proteins. Analysis of the *SHPK* transcript in cells of patient 1 or the mother showed no signs of nonsense-mediated decay. However, absence of SHPK protein in patient 1 and the strongly reduced SHPK activity in fibroblast homogenates of the patients indicated a severe SHPK deficiency.

The c.355C > T heterozygous allele variant has been detected in 8/4,300 European American and in 0/2,203 African American control individuals (rs144071313; http://evs.gs.washington.edu/EVS/). This relative high frequency of the heterozygous variant in controls suggests that SHPK deficiency is more common than currently known or that homozygosity of this variant is not associated with an inborn error of metabolism with a severe phenotype. The c.211G > T; p.Glu71X variant is a novel variant not previously reported.

There remains the question of whether SHPK deficiency is the causal factor for the clinical phenotypes of our patients. Although other causes for the clinical presentation were extensively investigated in both cases, we cannot exclude the presence of other recessive disorders in these consanguineous heritages. Since both patients presented very differently and without a clear clinical overlap with cystinosis patients (caused by the 57-kb deletion), the biochemical defect of SHPK deficiency is either unrelated to the clinical phenotypes or might have a broad phenotypic presentation, which is dependent on external factors and/or the genetic backgrounds of the individuals. The importance of SHPK as a regulatory enzyme has to be further investigated.

With high-throughput sequencing technologies known as next-generation sequencing (NGS), such as whole-exome (WES) and whole-genome (WGS) sequencing, more genetic variants are being detected. These variants have yet to undergo intensive analysis to determine whether they are indeed clinically relevant. In the past, frameshifts and nonsense mutations were considered pathogenic. This study illustrates that even if the mutation results in biochemical deficiency, it is questionable whether this gene is indeed the cause of the disease. Thus, not only functional studies but also extensive clinical workups are needed.

Clinical relevance of SHPK deficiency is currently unknown. Identifying other individuals with SHPK deficiency is needed for further clarification.

## Electronic supplementary material

Below is the link to the electronic supplementary material.

ESM 1 (DOC 58 kb)
