# Supplementary material for: First two unrelated cases of isolated sedoheptulokinase deficiency: A benign disorder?
Source: J Inherit Metab Dis. 2015 Feb 3;38(5):889–94. doi: 10.1007/s10545-014-9809-1 (PMC4551550; doi:10.1007/s10545-014-9809-1)
Supplement: Supplementary file 1 — (DOC 58 kb) [file 10545_2014_9809_MOESM1_ESM.doc]

Supplement table 1: Primers SHPK

| **genomic DNA** | **PCR**  **primers** | **Approximate size** | **Primer sequence*** |
| --- | --- | --- | --- |
| **exon 1** | DM1996 | 400 bp | GTAAAACGACGGCCAGGACGAGAAGCCTCCATGTTG |
|  | DM1997 |  | CAGGAAACAGCTATGAAGAGACTTCATTGCGGGAAG |
| **exon 2** | DM1998 | 360 bp | GTAAAACGACGGCCAGAGTTTGCTGCAACTCACCTG |
|  | DM1999 |  | CAGGAAACAGCTATGACGCACAGCACTGCTCTAGTT |
| **exon 3** | DM2000 | 370 bp | GTAAAACGACGGCCAGGGTGTGCCAGGCTTTATCAT |
|  | DM2001 |  | CAGGAAACAGCTATGACGGCCTATTCTCATTCTCCC |
| **exon 4** | DM2002 | 290 bp | GTAAAACGACGGCCAGGCACATGCTTCCGAGTTCAT |
|  | DM2003 |  | CAGGAAACAGCTATGAAAAGCACCCACTGAAGCACT |
| **exon 5** | DM2004 | 300 bp | GTAAAACGACGGCCAGTCCCTTAAATTCCCGAAACC |
|  | DM2005 |  | CAGGAAACAGCTATGAAGAGCTGGTGCTTATGAGGC |
| **exon 6** | DM2006 | 400 bp | GTAAAACGACGGCCAGTGACCTCTGAGTTAGCCCGA |
|  | DM2007 |  | CAGGAAACAGCTATGATGGGAGAAATATGCCAGGAC |
| **exon 7** | DM2008 | 590 bp | GTAAAACGACGGCCAGATTCTCTGCCATGTCAGCCT |
|  | DM2009 |  | CAGGAAACAGCTATGATGAAAGCTGTCCACTGAACG |

*Primers contain M13 forward (red) and reverse (green) sequences.
